# RETRACTION

**DOI:** 10.1590/1678-77572016retr002

**Published:** 2016

**Authors:** Karin Hermana Neppelenbroek

The Editorial Board of the Journal Applied Oral Science communicates the formal retraction of the manuscript:

Cintra LTA, Benetti F, Ferreira LL, Rahal V, Ervolino E, Jacinto RC, et al . Evaluation of an experimental rat model for comparative studies of bleaching agents. J Appl Oral Sci. 2016;24(2):171-80. http://dx.doi.org/10.1590/1678-775720150393

Since it comprises a duplicated version of a manuscript previously published in the preceding edition of the Journal Applied Oral Science:

Cintra LTA, Benetti F, Ferreira LL, Rahal V, Ervolino E, Jacinto RC, et al . Evaluation of an experimental rat model for comparative studies of bleaching agents. J Appl Oral Sci. 2016;24(1):95-104. http://dx.doi.org/10.1590/1678-775720150393.

Prof. Dr. Karin Hermana Neppelenbroek

Editor-in-Chief

